# Contrasting a Misinterpretation of the Reverse Contrast

**DOI:** 10.3390/vision4040047

**Published:** 2020-11-02

**Authors:** Tiziano Agostini, Mauro Murgia, Fabrizio Sors, Valter Prpic, Alessandra Galmonte

**Affiliations:** 1 Department of Life Sciences, University of Trieste, 34100 Trieste, Italy; mmurgia@units.it (M.M.); fsors@units.it (F.S.); 2 Department of Medicine, Surgery and Health Sciences, University of Trieste, 34100 Trieste, Italy; agalmonte@units.it; 3 Institute for Psychological Science, De Montfort University, Leicester LE1 9BH, UK; valter.prpic@dmu.ac.uk

**Keywords:** lightness, reverse contrast, belongingness, grouping, contrast, assimilation

## Abstract

The reverse contrast is a perceptual phenomenon in which the effect of the classical simultaneous lightness contrast is reversed. In classic simultaneous lightness contrast configurations, a gray surrounded by black is perceived lighter than an identical gray surrounded by white, but in the reverse contrast configurations, the perceptual outcome is the opposite: a gray surrounded by black appears darker than the same gray surrounded by white. The explanation provided for the reverse contrast (by different authors) is the belongingness of the gray targets to a more complex configuration. Different configurations show the occurrence of these phenomena; however, the factors determining this effect are not always the same. In particular, some configurations are based on both belongingness and assimilation, while one configuration is based only on belongingness. The evidence that different factors determine the reverse contrast is crucial for future research dealing with achromatic color perception and, in particular, with lightness induction phenomena.

Achromatic color perception concerns the ability of visual system to retrieve from luminance two variables present in the physical world, namely light intensity and reflectance, which at the phenomenal level are known as brightness and lightness. The visual system can only access the luminance, and from this information is able to attribute brightness and lightness, using relational factors and scene interpretation to constrain and solve the under-determined problem of retrieving the distal causes [1,2].

Within the lightness domain, the induction phenomena—contrast and assimilation—have been widely studied. The first one is a perceived increase of the difference between the lightness of the target and that of inducing regions, the second one is the opposite. These phenomena are traditionally accounted for in terms of low-level versus high-level processing (e.g., [3,4,5,6,7,8,9,10,11,12]). The low-level account is based on evidence on lateral inhibition [13,14], suggesting that contrast effects are due to the inhibition sent by the receptors stimulated by the light inducing regions to the receptors stimulated by the induced target. This neural mechanism would increase the lightness difference between the light background and the target (which appears darker). The high-level account is based on the principles of perceptual organization [12,15,16]. According to this account, the lightness of a target would be due to the global contrast between the target and the achromatic color of other surfaces to which it perceptually belongs.

Nevertheless, the debate is not solved, and novel theories attempted to explain lightness perception using different approaches [2,5,17,18,19,20]. New theories try to find an explanation that can be applied to the majority of phenomena known in the literature, and it is important that the mechanisms underlying the phenomena are not confounded. For this reason, in the present work we would like to point out some specific properties of different types of the *reverse (or reversed) contrast illusion* [21,22,23,24,25,26], which in previous studies have been erroneously considered equivalent. This phenomenon refers to the inversion of the classical simultaneous lightness contrast effect, and was observed in different displays.

In a recent paper by Economou, Zdravkovic and Gilchrist [27], the authors report that they parametrically manipulated the belongingness (see [28]) of two gray targets using several variations of the 2002 display by Gilchrist and Annan (see Figure 1a). The authors reported that their manipulation of grouping affects the size and direction of the illusion, giving rise to different examples of the reverse contrast illusion.

It is undeniable that in the displays by Economou, Zdravkovic and Gilchrist [27] the grouping factors have a role in determining the reverse contrast illusions; however, in our opinion, the spatial frequency factor is crucial for determining the effect. Indeed, Agostini, Murgia and Galmonte [25] demonstrated that the display by Gilchrist and Annan [21], as well as other similar displays (see [22]), do not show a “pure” reverse contrast illusion due to grouping factors, since the elimination of the grouping inducers do not determine the return to the simultaneous lightness contrast effect (see Figure 1b,c). The only exception is the display by Agostini and Galmonte (see Figure 2a), first presented at The Association for Research in Vision and Ophthalmology (ARVO) annual meeting [23] and then published in Psychological Science [24], in which the elimination of the grouping inducers determines the inversion of the effect (see Figure 2b). Therefore, it appears evident that in Gilchrist and Annan display [21], as well as in its variations (including those of Economou, Zdravkovic, and Gilchrist [27]), there is the effect of a confounding variable that is salient in the perceptual outcome of reverse contrast. To deepen our reasoning, it is necessary to take a step backward and further analyze both Agostini and Galmonte [24] and Gilchrist and Annan [21] displays.

Agostini and Galmonte [24] created a novel lightness configuration by modifying a Necker cube, which served to guarantee the intervention of global grouping factors. In their display, one cube is made by middle-gray dashed lines and dark corners, and is placed on a light background; while the second one is the photographically reversed version of the first one, being made by middle-gray dashed lines and light corners placed on a dark background (Figure 2a). In this configuration, the local and global inducers compete: the perceived color of the dashed lines is both contrasted by the background (because of local lateral inhibition), and by the corners (because of global belongingness/grouping to the cube), but the direction of the effects is reversed. Indeed, if local contrast prevailed, the dashed lines should be perceived as in the classical simultaneous lightness contrast, being the effect elicited by the background. If global contrast (belongingness/grouping) prevailed, the color of the dashed lines should be perceived as the reverse of simultaneous lightness contrast, being the effect elicited by the belongingness/grouping to the corners. The results clearly demonstrated a superiority of global belongingness: the dashed lines locally surrounded by the light background appear lighter than those surrounded by the dark background (and vice versa for the other cube), thus reversing the classical simultaneous lightness contrast.

Gilchrist and Annan [21] proposed another configuration of reverse contrast, in which several parallel black strips are surrounded by white, and several parallel white strips are surrounded by black. Two gray strips, equal to the black/white strips in terms of dimension, orientation, and distance from the other strips, are placed in the middle of the parallel black/white strips, thus being totally surrounded by the white/black background (Figure 1a). In this case, perceptual belongingness is determined by good continuation, proximity, and shape similarity. The gray aligned with the black strips is perceived as lighter than the other, although it is locally surrounded by white. Thus, also this result seems to work in favor of perceptual grouping instead of local contrast.

Even though the Agostini and Galmonte [24] and Gilchrist and Annan [21] displays appear to be only two different versions of the same phenomenon, actually they are based on different factors. During the 24th ECVP conference [30], two of us demonstrated that the Agostini and Galmonte’s effect is based only on the grouping factor, while the Gilchrist and Annan’s configuration is based on both grouping and spatial frequency factors (both configurations had been previously presented in international meetings on perception, at the end of the 1990s). This evidence has been largely ignored by the scientific community for years; indeed, in most of literature, the reverse contrast illusion elicited by these displays has been considered to be the outcome of the same mechanisms, as claimed also by Economou, Zdravkovic, and Gilchrist [27]. This fact has determined a damage for research in the domain of lightness, since theories and models have wrongly used one single explanation for both displays, based on their supposed equivalence [27,31,32].

To avoid that other scientists would continue misinterpreting the factors underpinning the different displays of the reverse contrast, in 2014 three of us published a paper titled “Reversing the reversed contrast” in which we empirically demonstrated that the above-mentioned configurations are based on different mechanisms [25]. Participants were required to judge the lightness of the gray targets of the original and of some modified versions of the above-cited configurations, in which grouping factors were removed. If the grouping factors were the only responsible for the reverse contrast, then the perceptual outcome should be reversed. The results showed that the effect was reversed only in the Agostini and Galmonte’s configuration (see Figure 2b), while it did not happen in the Gilchrist and Annan’s configuration (see Figure 1b,c), as well as in the Bressan’s configuration [22]. This suggests that the factors determining the Agostini and Galmonte’s effect are different from those acting on the Gilchrist and Annan’s configuration, in which the lightness change is due also to factors other than belongingness. We concluded that in Gilchrist and Annan’s configuration the physiological principle of spatial summation plays a role in determining the overall effect; indeed, high spatial frequencies give rise to assimilation phenomena [29]. 

Our conclusion on Gilchrist and Annan’s configuration is consistent with several qualitative results demonstrating that assimilation occurs mainly with narrow surrounds [29,33,34]. The quantitative results of Rudd [35] are in the same direction. In disk/annulus displays, the author found contrast when the annulus was sufficiently wide, while he observed assimilation with narrower annuli. Since disks and annuli were always on the same remote background, it could not have been the factor determining assimilation. This situation can be considered somewhat similar to those studied first by Gilchrist and Annan [21] and then by Economou, Zdravkovic and Gilchrist [27].

Based on our previous work [25], we can claim that in Gilchrist and Annan’s display, as well as in Economou, Zdravkovic and Gilchrist ones, the effect on the grays is the result of the combination of at least three factors: (1) the color of the strips to which they belong (black/white strips induce lightening/darkening, meaning a contrast effect); (2) the color of the overall background (white/black background induces darkening/lightening, meaning a contrast effect); (3) the color of the flanking regions (white/black flanking regions induce lightening/darkening, meaning an assimilation effect). In all the seven experiments reported in Economou, Zdravkovic and Gilchrist [27] all these factors are still contemporaneously present and they are not separately manipulated. Thus, even though this fact does not detract at all the interest and the value of their results, it is anyway necessary to be aware that they are not studying a “pure” reverse contrast effect due to grouping factors. Indeed, their effect is determined not only by grouping/belongingness factors, but also by the co-varying variable of assimilation, which arises from the use of high spatial frequency configurations.

If a researcher is interested in understanding more deeply the grouping factors involved in the reverse contrast phenomenon, s/he should use what in research methods is called the most suitable preparation as possible, i.e., the one that both leads to the stronger effect and that has less as possible co-varying/confounding variables. In White and McBurney’s words [36] (p. 197): *“[…] one of the researcher’s goal is to choose the most suitable preparation for studying a given problem. Some of the most important contributions to psychology have been made by people who selected an appropriate preparation”*. In this case, due to the less confounding variables, the configuration of Agostini and Galmonte [24] would be the “most suitable preparation” for studying the grouping factors in the reverse contrast illusions. Therefore, from both methodological and theoretical perspectives, it is important for researchers to be aware of the actual differences between the above-mentioned displays. As a consequence, it is necessary that researchers do not consider the perceptual outcome of these different configurations as the result of a single phenomenon (as assumed by different authors: [27,31]), and do not automatically extend the explanation of one configuration to the other.

## Figures and Tables

**Figure 1:**
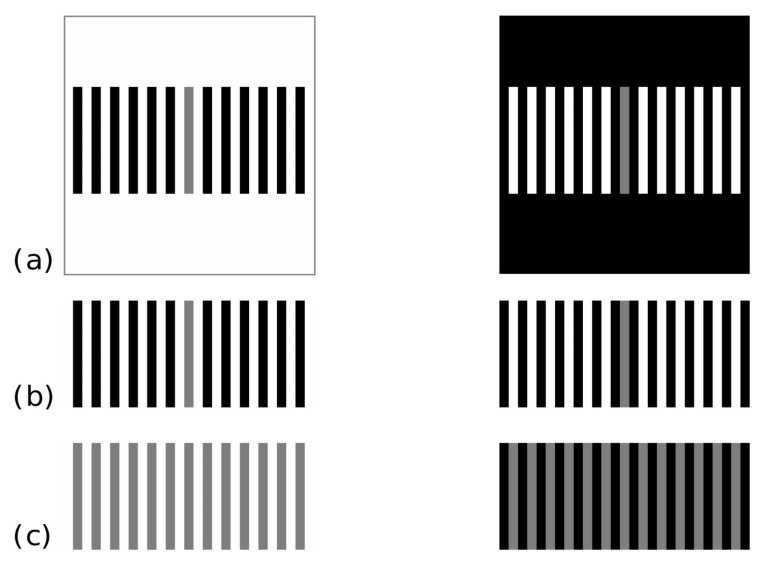
(**a**) Gilchrist and Annan’s [21] original reversed contrast configuration; (**b**) same configuration with a reduced background: removing the global belongingness/grouping factor, the effect is still that of reversed lightness contrast, as modified by Agostini et al. [25]; (**c**) same configuration with reduced background and with only gray strips: this is the classical assimilation configuration by Helson [29].

**Figure 2:**
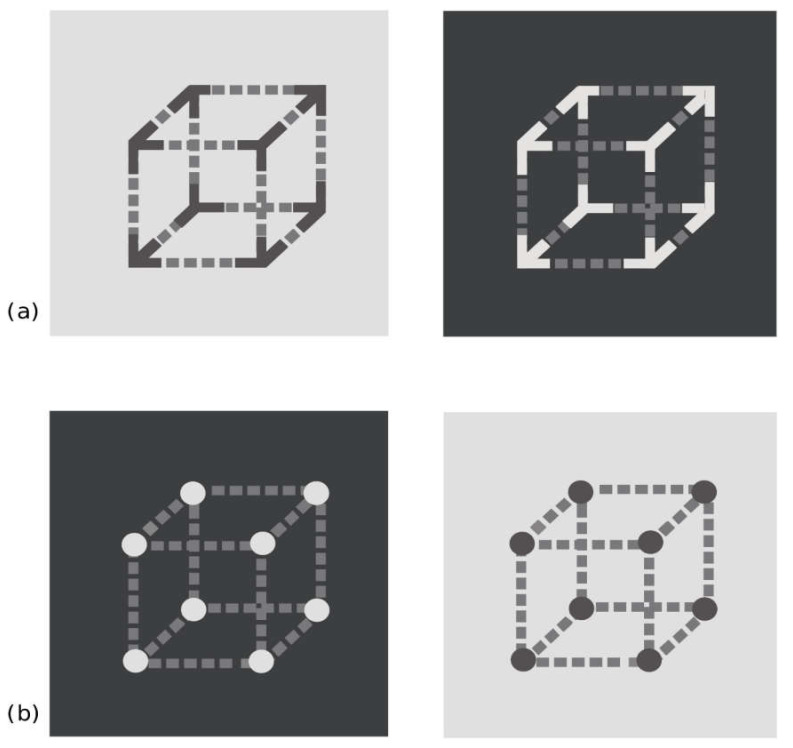
(**a**) Agostini and Galmonte [24] original reversed contrast Necker Cube; (**b**) same configuration with occluding disks instead of the inducer corners (control condition): removing the global belongingness/grouping factor, the effect is inverted to simultaneous lightness contrast.
